# A qualitative study of patient choices between herbal and hospital care for infectious diseases in Somalia

**DOI:** 10.1038/s41598-025-11167-8

**Published:** 2025-09-30

**Authors:** Mohamed Jayte, Abdifitah Abdullahi Mohamed, Abishir Mohamud Hersi, Yahye Mohamed Jama, Awil Abdulkadir Abdi, Ibrahim Ahmed Nor

**Affiliations:** 1 Internal Medicine Department at Kampala International University, P.O. Box 7062, Kampala, Uganda; 2 Microbiology Department at Kampala International University, Kampala, Uganda

**Keywords:** Traditional medicine, Infectious diseases, Healthcare access, Somalia, Trust, Antibiotic resistance, Qualitative study, Biochemistry, Biological techniques, Microbiology

## Abstract

Somalia’s healthcare system is marked by a reliance on traditional medicine and under-resourced modern facilities. This study explores how communities navigate treatment choices for infectious diseases, such as malaria and cholera, amid competing trust in herbal remedies and biomedical care. We conducted in-depth interviews with 30 participants, including 15 patients, 10 traditional healers, and 5 healthcare providers, alongside 3 focus group discussions (FGDs). Data were analyzed using thematic analysis. Three key themes emerged: (1) the cultural legitimacy of traditional medicine, (2) the perceived efficacy and safety of herbs versus antibiotics, and (3) structural barriers to accessing hospitals. Participants expressed divergent perspectives, with some preferring hospitals for severe infections due to perceived reliability, while others relied on herbal medicine due to financial constraints and cultural alignment. Somalia’s healthcare challenges require integrating traditional and modern medicine while addressing structural barriers. Key solutions include training traditional healers, implementing health subsidies, and launching community education on proper medication use. By combining these approaches, Somalia can build a culturally sensitive, accessible healthcare system that improves public health outcomes.

## Introduction

Somalia’s healthcare system operates within a challenging environment characterized by decades of conflict, political instability, and limited infrastructure. Infectious diseases such as malaria, cholera, and respiratory infections remain leading causes of morbidity and mortality, with the country ranking among the highest in disease burden in sub-Saharan Africa^1^. Compounding this issue is the dual reliance on traditional herbal medicine and under-resourced modern healthcare facilities. While traditional medicine is deeply rooted in Somali culture and history, the biomedical system struggles to meet the population’s needs due to chronic underfunding and inadequate staffing^2^.

Traditional medicine in Somalia is not merely an alternative form of healthcare but a cornerstone of cultural identity. Herbal remedies are often perceived as more accessible, affordable, and aligned with local beliefs compared to modern treatments^3^. However, this reliance on traditional medicine can lead to delays in seeking biomedical care, particularly for infectious diseases that require timely intervention. Furthermore, the lack of integration between traditional and modern healthcare systems exacerbates fragmentation, resulting in suboptimal health outcomes^4^.

A critical gap in the literature is the lack of data on how trust influences treatment decisions in Somalia. Trust is a multifaceted construct that encompasses cultural legitimacy, perceived efficacy, and accessibility. While some studies have explored the use of traditional medicine in other African contexts, few have examined the Somali experience, particularly in relation to infectious diseases^5^.

Religion, particularly Islam, plays a significant role in shaping healthcare perceptions and practices in Somalia. Traditional medicine is often intertwined with Islamic practices, such as the use of Quranic verses or prayers alongside herbal remedies. Many Somalis view traditional medicine as divinely inspired, reinforcing its cultural and spiritual legitimacy^8^. This religious dimension complicates efforts to promote modern healthcare, as hospitals and biomedical treatments may be perceived as secular or foreign. Understanding the interplay between religion and healthcare is essential for designing culturally sensitive interventions that respect local beliefs while promoting safe and effective treatments.

This study aims to address these gaps by investigating the factors that shape preferences for traditional versus modern care in Somalia. Specifically, it explores the cultural, structural, and economic dimensions of healthcare decision-making and proposes strategies for integrating traditional and biomedical systems. By doing so, this research contributes to the broader discourse on healthcare access and equity in conflict-affected settings.

## Methods

### Study design and setting

This qualitative exploratory study employed semi-structured interviews and focus group discussions (FGDs) to explore community perceptions of traditional and modern healthcare for infectious diseases. The study was conducted in **Mogadishu**,** Somalia**, from **November to December 2024**. Mogadishu was chosen due to its diverse population, availability of both traditional healers and modern healthcare facilities, and high burden of infectious diseases.

### Participant selection and sample size justification

Participants were purposively selected to include the following groups:

- **Patients** (*n* = 15): Individuals aged **25 years or older** who had used either traditional herbs or hospitals for treating malaria or cholera within the past year.
- **Traditional healers** (*n* = 10): Practitioners with at least five years of experience in herbal medicine.
- **Healthcare providers** (*n* = 5): Medical doctors and nurses working in public or private hospitals.

The sample size of **30 participants** was determined based on the principle of **thematic saturation**, where no new themes emerged after approximately **25 interviews and 2 FGDs**. This number is consistent with qualitative research standards for achieving depth and diversity in participant perspectives^7^.

### Data collection

Data were collected through **in-depth interviews** and **3 FGDs**. One of the authors, fluent in Somali, conducted all interviews in the local language to ensure cultural and linguistic sensitivity. Interviews were audio-recorded with participants’ **verbal consent** and later transcribed verbatim. Topics included decision-making processes, trust in treatments, experiences with stigma, and financial barriers. Each interview lasted approximately 45–60 min, while FGDs lasted 90–120 min.

**Interview guide**:

The interview guide included open-ended questions such as:

- What factors influence your choice between traditional herbs and hospitals?
- How do you perceive the effectiveness and safety of herbal remedies versus antibiotics?
- What barriers prevent you from accessing hospitals?
- How do you perceive doctors compared to herbalists?
- What role does family or religion play in your healthcare decisions?
- Have you ever had a negative experience with herbal or hospital treatments?

### FGD structure

FGDs were stratified by participant type to facilitate open discussion, ensuring that patients, traditional healers, and healthcare providers could freely share their perspectives without hierarchy-induced bias. Specifically, one FGD was conducted with patients, one with traditional healers, and one with healthcare providers.

### Translation process

All interviews were conducted in Somali and later translated into English for analysis. The translation process was carefully managed to ensure accuracy and preserve cultural nuances. A bilingual researcher, fluent in both Somali and English, reviewed the transcripts to verify the fidelity of the translations. Special attention was given to preserving idiomatic expressions and culturally specific terms to maintain the richness of the data.

### Data analysis

Thematic analysis was conducted using **NVivo software**. Transcripts were coded inductively, and themes were developed through an iterative process. Initial codes were grouped into categories, which were then refined into overarching themes. To ensure rigor, **two researchers independently coded the data**, and discrepancies were resolved through **peer debriefing**. Member checking was also performed by sharing preliminary findings with participants for validation.

## Results

The study included 30 participants, comprising 15 patients, 10 traditional healers, and 5 healthcare providers, as detailed in Table 1. The participants represented diverse demographics, with patients ranging from 25 to 50 years old, traditional healers from 35 to 65, and healthcare providers from 30 to 55. Educational levels varied, with most patients having primary or no formal education, traditional healers relying on informal training, and healthcare providers holding tertiary qualifications. Socioeconomic status also differed, with the majority of patients and traditional healers identifying as low-income, while healthcare providers were middle-income.

Three main themes emerged from the data, as summarized in Table 2: (1) the cultural legitimacy of traditional medicine, (2) perceived efficacy and mistrust in modern care, and (3) structural barriers to accessing hospitals. Participants emphasized the cultural significance of herbal remedies, viewing them as ancestral wisdom and a symbol of Somali identity. However, skepticism about antibiotics and the high cost of hospital care were significant barriers to accessing modern healthcare. These findings highlight the complex interplay between cultural trust, perceived efficacy, and structural challenges in healthcare decision-making in Somalia.

**Table 1:** Characteristics of the study participants.

Category	Number	Gender (M/F)	Age range	Education level	Socioeconomic status	Role/profession
Patients	15	8 M / 7 F	25–50	Primary (10), None (5)	Low-income (12), Middle-income (3)	N/A
Traditional Healers	10	6 M / 4 F	35–65	Informal (10)	Low-income (8), Middle-income (2)	Traditional Healer
Healthcare Providers	5	3 M / 2 F	30–55	Tertiary (5)	Middle-income (5)	Medical Doctor (3), Nurse (2)

**Table 2:** Summary of themes, categories, and subcategories.

Theme	Category	Subcategory
1. Cultural Legitimacy of Traditional Medicine	Cultural Identity	Herbs as ancestral wisdom
		Herbs as a symbol of Somali identity
	Accessibility and Affordability	Herbs as first-line treatment
		Financial constraints in modern care
2. Perceived Efficacy and Mistrust in Modern Care	Skepticism about Antibiotics	Belief in herbal “completeness”
		Fear of antibiotic side effects
	Trust in Modern Healthcare	Healthcare providers’ perspectives
3. Structural Barriers to Hospital Access	Financial Constraints	High cost of hospital care
		Lack of insurance or subsidies
	Stigma and Discrimination	Fear of being labeled “foreign”
		Community disapproval of hospitals

***Theme 1: Cultural Legitimacy of Traditional Medicine***

This theme highlights the deep cultural roots of traditional medicine in Somalia and its role as a symbol of identity and continuity. Participants from all groups emphasized the cultural significance of herbal remedies, though their perspectives varied based on their roles and experiences.

**Subcategory 1.1: Herbs as Ancestral Wisdom**

Traditional healers and patients alike described herbal medicine as a legacy passed down through generations. A traditional healer explained:

*“Herbs are our ancestors’ wisdom. They have been used for centuries to treat diseases. Hospitals are for emergencies*,* but herbs are for everyday healing.”* (Healer, 52).

**Subcategory 1.2: Herbs as a Symbol of Somali Identity**

Participants frequently described herbal medicine as a marker of Somali identity. A 28-year-old patient noted:

*“When I use herbs, I feel connected to my ancestors and my culture. Hospitals feel foreign, like they belong to someone else.”*

**Subcategory 1.3: Herbs as First-Line Treatment**

Many participants viewed herbs as the first option for treating illnesses. A traditional healer stated:

*“Most people come to me first. They only go to the hospital if the herbs don’t work.”* (Healer, 60).

**Subcategory 1.4: Gender Roles and Traditional Medicine**

Gender played a significant role in shaping preferences for traditional medicine. Women, particularly in rural areas, were more likely to rely on herbs due to cultural expectations and limited mobility. A 30-year-old female patient explained:

*“As a woman, it’s easier for me to use herbs. Going to the hospital requires permission and transportation, which I often don’t have.”*

***Theme 2: Perceived Efficacy and Mistrust in Modern Care***

This theme explores participants’ skepticism about the efficacy and safety of modern treatments, particularly antibiotics.

**Subcategory 2.1: Belief in Herbal “Completeness”**.

Patients and traditional healers frequently described herbal remedies as more “complete” or holistic than modern treatments. A 42-year-old patient shared:

*“Herbs treat the whole body, not just the disease. They cleanse your blood and strengthen your spirit. Antibiotics only target one thing.”*

**Subcategory 2.2: Fear of Antibiotic Side Effects**.

A recurring concern among patients was the belief that antibiotics weaken the body. A 34-year-old patient explained:

*“Antibiotics make you weak; herbs cleanse the body. When I take antibiotics, I feel tired and sicker than before.”*

**Subcategory 2.3: Role of Religion in Shaping Perceptions**.

Religion, particularly Islam, influenced perceptions of modern medicine. Many participants viewed traditional medicine as divinely inspired, reinforcing its cultural and spiritual legitimacy. A traditional healer noted:

*“Herbs are blessed by Allah. They are part of our faith and our way of life. Hospitals are man-made*,* but herbs are a gift from God.”* (Healer, 55).

**Subcategory 2.4: Choosing Herbs Over Antibiotics for Infections**.

Participants commonly reported choosing herbal remedies over antibiotics, even for infections like malaria or diarrhea. A 33-year-old patient noted: *‘I use herbs first because they work without causing side effects. Antibiotics make me feel worse before I feel better*.’ Another participant added, *‘I avoid antibiotics because they are too strong. Herbs are natural and gentle*.‘”.

### Healthcare providers’ perspectives

Healthcare providers expressed frustration with the mistrust of modern treatments. A medical doctor stated:

*“Many patients come to us too late because they tried herbs first. We need to educate people about the risks of delaying treatment.”* (Medical Doctor, 45).

A nurse added:

*“We see patients who have been misled by traditional healers about the dangers of antibiotics. It’s a constant battle to build trust in modern medicine.”* (Nurse, 38).

**Subcategory 2.5: Interest in Combining Herbal and Hospital Treatments**.

While not explicitly a dominant theme, a few participants mentioned the possibility of using both traditional and hospital treatments. A 31-year-old male patient shared: ‘Sometimes I use herbs to start, then go to the hospital if it doesn’t work. Both have their benefits.’ Another participant suggested, ‘Doctors should understand our herbs too; they should work together with healers.’ These comments suggest a growing openness among community members toward a more integrative model of care.”

***Theme 3: Structural Barriers to Accessing Hospitals***.

This theme highlights the practical challenges that deter individuals from seeking hospital care.

**Subcategory 3.1: High Cost of Hospital Care**.

The high cost of hospital care was a major barrier for many patients. A 25-year-old patient stated:

*“I cannot afford hospital fees. Herbs are my only option. Even if I wanted to go to the hospital, I don’t have the money.”*

**Subcategory 3.2: Geographic Barriers**.

Distance to the nearest hospital was a significant deterrent, particularly for rural residents. A 40-year-old patient from a rural area shared:

*“The nearest hospital is hours away, and I don’t have the means to travel. Herbs are always available in my village.”*

**Subcategory 3.3: Lack of Insurance or Subsidies**.

Participants frequently cited the lack of insurance or government subsidies as a barrier. A traditional healer noted:

*“If the government provided free healthcare*,* more people would go to hospitals. But right now*,* herbs are the only option for many.”* (Healer, 60).

## Discussion

The findings of this study demonstrate the complex relationship between cultural trust, perceived effectiveness, and structural barriers in healthcare decision-making in Somalia. Traditional medicine remains strongly connected to Somali cultural identity, with many participants viewing herbal remedies as representations of ancestral knowledge and cultural heritage. This cultural connection creates challenges for promoting biomedical care, as modern healthcare facilities are often seen as unfamiliar or difficult to access^9^. Comparable patterns have been documented in other resource-limited settings including Ethiopia and Nigeria, where traditional medicine maintains popularity due to its affordability and cultural compatibility^10^. These persistent attitudes emphasize the necessity for healthcare approaches that honor traditional practices while working to improve the existing system.

A primary finding of this research is the prevalent antibiotic avoidance stemming from misinformation and distrust. Numerous participants believed antibiotics could harm the body’s natural strength, while considering herbal treatments as more balanced and harmonious with the body’s systems. These views correspond with worldwide patterns of antibiotic avoidance, especially in areas with limited health education^11^. Research in Kenya and Tanzania has recorded similar misunderstandings about antibiotics, with many people considering them potentially dangerous or only needed for extreme symptoms^12^. Community healers, who serve as important healthcare providers, frequently have no training about proper antibiotic use, which worsens the situation^11^. Solving this problem demands immediate efforts to connect traditional and modern medical systems for safer, more effective healthcare practices.

Additionally, there is limited research on community perceptions of antibiotic resistance, a growing global health threat that is particularly acute in low-resource settings like Somalia^1^.

In Somalia, the preference for traditional medicine over modern pharmaceuticals remains prevalent, particularly in rural and underserved communities. Many individuals seek care from traditional healers due to cultural beliefs, accessibility, and affordability. As a result, antibiotics are often avoided, underused, or misunderstood, with some healers incorporating them into herbal remedies without adequate knowledge of their proper indications, dosage, or duration^6^. This tendency to prioritize traditional treatments can delay appropriate medical intervention, leading to prolonged illness or complications. Addressing this issue requires culturally sensitive public health strategies, including education on the appropriate use of antibiotics, regulation of both pharmaceutical and traditional health practices, and improved collaboration between traditional healers and biomedical practitioners^7^.

The cultural reluctance toward antibiotic use in Somalia represents a serious public health concern. Within traditional healing practices, antibiotics are occasionally added to herbal preparations without correct understanding of proper amounts or treatment length. Moreover, fake antibiotics commonly found in unofficial markets decrease confidence in their effectiveness^13^. Parallel difficulties have been noted in other African nations like Ghana and Uganda, where uncontrolled antibiotic distribution and incorrect use in traditional medicine contribute to avoidance behaviors^14^. Confronting this issue requires comprehensive solutions, including educational initiatives for traditional healers about appropriate antibiotic use and the value of directing patients to hospitals when necessary^14^.

Combining traditional and modern healthcare approaches is crucial for enhancing health results in Somalia. Traditional healers, who enjoy substantial community trust and availability, could be instrumental in this merging of systems. A possible method would involve training healers as community health workers, allowing them to deliver basic medical services like wound treatment and health information while sending more serious cases to hospitals^1,13^. Creating joint clinics providing both traditional and biomedical treatments might improve working relationships between healers and doctors. Additionally, implementing organized referral systems would help guarantee patients obtain suitable care regardless of whether they first consult a healer or hospital. Comparable integration attempts have shown success in nations such as South Africa and India, where including traditional healers in official healthcare systems has produced better health outcomes and increased public confidence in modern medicine^15^.

This investigation has certain limitations, including a restricted number of participants and possible inaccuracies in self-reported information. Subsequent research should involve larger sample groups and examine long-term patterns in healthcare choices. Further studies might also assess how well integration efforts between traditional and modern healthcare systems work, along with evaluating educational programs about antibiotic avoidance and healthcare availability.

## Conclusion

Somalia’s strong cultural preference for traditional medicine, combined with structural barriers to healthcare access, requires integrated solutions. Key recommendations include: (1) incorporating traditional healers into community health programs, (2) implementing culturally appropriate health education, and (3) reducing financial barriers through subsidies. Addressing antibiotic avoidance is critical, particularly regarding improper use in traditional practices and counterfeit medications. Improving healthcare access requires tackling cost, distance, and stigma through infrastructure development, insurance schemes, and trust-building initiatives. By thoughtfully combining traditional and modern approaches, Somalia can build a more inclusive, effective healthcare system that respects cultural values while improving health outcomes.

## Data Availability

Data are available upon reasonable request from the corresponding author, Mohamed Jayte (maxamedjeyte@gmail.com).
